# Thermal Stability and Flammability Studies of MXene–Organic Hybrid Polystyrene Nanocomposites

**DOI:** 10.3390/polym14061213

**Published:** 2022-03-17

**Authors:** Zhuoran Zhang, Huaixuan Cao, Yufeng Quan, Rong Ma, Emily B. Pentzer, Micah J. Green, Qingsheng Wang

**Affiliations:** 1 Artie McFerrin Department of Chemical Engineering, Texas A&M University, College Station, TX 77843, USA; zhuoranzhang@tamu.edu (Z.Z.); chx744933979@tamu.edu (H.C.); yquan@tamu.edu (Y.Q.); marong1863@tamu.edu (R.M.); micah.green@tamu.edu (M.J.G.); 2 Department of Materials Science and Engineering, Texas A&M University, College Station, TX 77843, USA; emilypentzer@chem.tamu.edu; 3 Department of Chemistry, Texas A&M University, College Station, TX 77843, USA

**Keywords:** pyrolysis-combustion flow calorimeter, flammability, thermal stability, polymer nanocomposite, MXene

## Abstract

Polystyrene (PS) is widely used in the plastics industry, but the application range of PS is limited due to its inherently high flammability. A variety of two-dimensional (2D) nanomaterials have been reported to impart excellent flame retardancy to polymeric materials. In this study, a 2D nanomaterial MXene–organic hybrid (O-Ti_3_C_2_) was applied to PS as a nanofiller. Firstly, the MXene nanosheets were prepared by acid etching, intercalation, and delamination of bulk MAX (Ti_3_AlC_2_) material. These exfoliated MXene nanosheets were then functionalized using a cationic surfactant to improve the dispersibility in DMF. Even with a small loading of functionalized O-Ti_3_C_2_ (e.g., 2 wt%), the resulting PS nanocomposite (PS/O-Ti_3_C_2_) showed good thermal stability and lower flammability evidenced by thermogravimetric analysis (TGA) and pyrolysis-combustion flow calorimetry (PCFC). The peak heat release rate (pHRR) was significantly reduced by 32% compared to the neat PS sample. In addition, we observed that the temperature at pHRR (T_pHRR_) shifted to a higher temperature by 22 °C. By comparing the TGA and PCFC results between the PS/MAX and different weight ratios of PS/O-Ti_3_C_2_ nanocomposites, the thermal stability and 2D thermal- and mass-transfer barrier effect of MXene–organic hybrid nanosheets were revealed to play essential roles in delaying the polymer degradation.

## 1. Introduction

Polystyrene (PS) is one of the most widely used thermoplastics in daily life (home appliances, decorations, packaging, etc.) due to its low density, natural transparency, ease of processing, and good chemical resistance [1,2,3]. However, because of its inherently high flammability, the application range of PS is limited for safety reasons [4]. The range of uses for PS can be expanded by improving its fire safety performance through the use of flame retardant (FR) additives [5,6].

Traditional halogenated FRs are gradually being phased out due to byproducts that are harmful to the environment and human health during combustion. In addition, the high loading (>30 wt%) of some halogen-free FRs, such as metal hydroxides, may deteriorate the mechanical and thermal properties of the polymer [7]. Recently, inorganic nanoparticles have attracted growing interest as novel FRs due to their impressive fire safety performance at a relatively low loading (<5 wt%) [8,9]. Several nanomaterials with two-dimensional (2D) layered structures, such as montmorillonite (MMT) [10], layered hydroxides (LDH) [11], and graphene [12], have shown promising flame retardancy in polymer nanocomposites by creating a “tortuous path” that delays pyrolysis products mixing with oxygen [13].

MXenes are a relatively new class of graphene-like 2D layered nanomaterials that have received considerable attention due to their excellent thermal stability, conductivity, and mechanical properties [14,15]. The chemical formula of MXenes is M_n+1_X_n_T_x_, where M represents an early transition metal element such as titanium (Ti), X is carbon (C) or nitrogen (N), and T stands for surface terminations. Due to the transition metal elements [16] and unique 2D structure, MXenes have the potential to be additives for preparing FR polymer composites. Furthermore, the dispersion of the 2D layered nanomaterial additives in the polymer has a significant effect on the performance of nanocomposites. The abundance of functional groups (e.g., -OH, =O, and -F) on the surface of MXenes makes it possible for them to be highly efficient FRs with excellent dispersion [17]. Recently, surface treatment of MXenes for polymeric nanocomposites with improved properties by various approaches has been developed [18,19]. Specifically, MXene-based FRs have been employed to improve the flame retardancy of polymers, including poly (vinyl alcohol) (PVA) [20,21], poly (lactic acid) (PLA) [22], thermoplastic polyurethane (TPU) [23], and unsaturated polyester resin (UPR) [24]. In addition, Si et al. fabricated exfoliated functionalized MXene PS nanocomposites with good FR properties via co-coagulation and compression molding [25]. They also investigated the flame retardancy and toxic gas products of the PS nanocomposites using a cone calorimeter. However, flammability characteristics of bench-scale tests such as cone calorimetry are dependent on experimental conditions as well as external physical factors [26,27]. Additional research on inherent material properties is needed to further develop MXenes as novel FRs in the polymer matrix.

Pyrolysis-combustion flow calorimetry (PCFC) has become an essential screening tool for the flammability and combustibility of polymers and their composites since introduced by Lyon and Walters in 2004 [28,29]. PCFC, also known as microscale combustion calorimeter (MCC), allows a researcher to evaluate the combustibility of a small polymeric sample (2–5 mg) under aerobic or anaerobic pyrolysis and complete combustion (ASTM D 7309). Through an oxygen analyzer, oxygen consumption and the heat release rate (HRR) can be calculated according to Huggett’s relation [30]. During a test, an HRR vs. temperature curve is recorded, along with heat release capacity (HRC, J/g∙K), peak heat release rate (pHRR, W/g), total heat release (THR, kJ/g), the temperature at pHRR (T_pHRR_, °C), and the heating rate (°C/s). Recent studies have combined heat-related PCFC and mass-related thermogravimetric analysis (TGA) to obtain additional flammability characteristics [29]. Using PCFC and TGA could further improve understandings of the combustion process of polymeric material and the polymer degradation mechanism. In this work, the flammability of the MXene–organic hybrid PS nanocomposites is investigated using PCFC and TGA and will give insight into the polymer degradation mechanisms of MXene-based nanocomposites.

## 2. Materials and Methods

### 2.1. Materials

Ti_3_AlC_2_ (MAX phases, ≥90%, ≤40 µm), hydrochloric acid (HCl, 37% [*w*/*w*], ACS reagent), dimethyl sulfoxide (DMSO, ≥99.5%), and polystyrene (PS, M_w_~192,000) were obtained from Sigma-Aldrich Inc., MO, USA. Octadecyl trimethyl ammonium bromide (OTAB, 99%) and *N*, *N*-dimethylformamide (DMF, certified ACS reagent, ≥99.8%) were obtained from Fisher Scientific Co., Hampton, NJ, USA. Lithium fluoride (LiF, 98%+) was purchased from Alfa Aesar, MA, USA.

### 2.2. Preparation of Titanium Carbide Ti_3_C_2_T_x_ (MXene) Nanosheet

The Ti_3_C_2_T_x_ MXene nanosheet was prepared according to our previous work. Briefly, a 20 mL 6M HCl solution was prepared, and 1.6 g of LiF was stirred into the solution. Then, 2 g of MAX powder was slowly added into the solution. The mixture was stirred at 40 °C for 40 h. Then, the resulting suspension was washed with DI water until the water effluent reached pH ~6. The sediment was then collected and dispersed in DMSO solution and stirred continuously for 20 h at room temperature. After intercalating with DMSO, the MXene clay was washed with DI water three times and then bath sonicated for 1 h. Finally, the resulting MXene suspension was centrifuged at 3500 rpm for 45 min, and the supernatant was collected as MXene nanosheets.

### 2.3. Modification of MXene–Organic Hybrid

As an organic cationic modifier, alkylammonium salt OTAB was inserted between the MXene layers through electrostatic adsorption. The supernatant obtained from centrifugation in the MXene synthesis was diluted to 1 mg/mL and ultrasonicated for 30 min under an ice bath. The suspension was stirred at room temperature for 30 min. Next, the suspension was washed with DI water and centrifuged (9000 rpm, 15 min) several times at room temperature to remove the unexchanged surfactants. Finally, the precipitate was collected and freeze-dried for at least 24 h to obtain MXene–organic hybrid (O-Ti_3_C_2_) powders.

### 2.4. Fabrication of PS/MAX, PS/MXene, and PS/MXene–Organic Hybrid Nanocomposite

PS/MXene–organic hybrid (PS/O-Ti_3_C_2_) nanocomposite was fabricated by the solution-mixing method. For example, 20 mg of O-Ti_3_C_2_ powders were dispersed in 8 mL DMF and ultrasonicated for 30 min under an ice bath. Then, 0.98 g of PS pellets were added to the O-Ti_3_C_2_/DMF dispersion with magnetic stirring. After all PS pellets were dissolved, the solution was slowly dropped into DI water to form pellet-shaped suspended particles. Finally, the products were dried in a room-temperature vacuum oven for 24 h to remove residual solvent. The content of O-Ti_3_C_2_ in PS nanocomposite was adjusted to create a range of 2/4/6 wt%.

The preparation methods for the PS/Ti_3_AlC_2_ and PS/Ti_3_C_2_ nanocomposite were similar to the PS/O-Ti_3_C_2_ nanocomposite, except for the type of additive used. The content of the MAX or MXene additive in PS nanocomposite was 2 wt%.

**Scheme 1:**
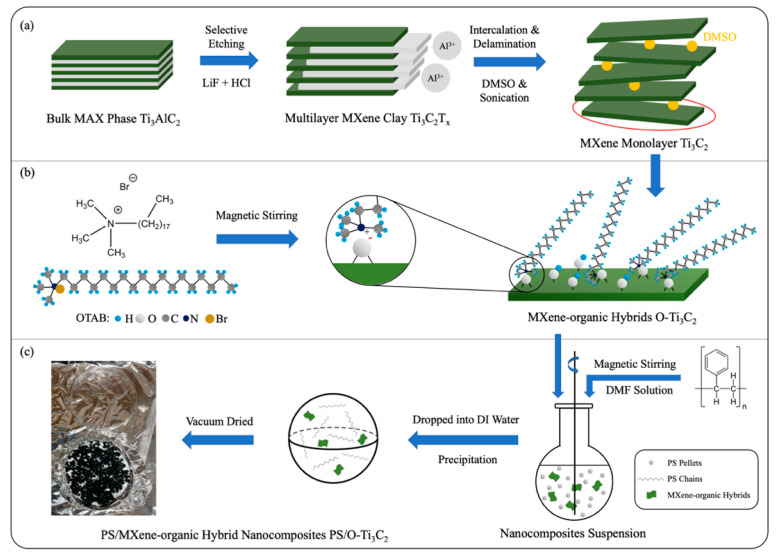
Illustration for preparation of (**a**) exfoliated Ti_3_C_2_ nanosheets, (**b**) functionalized O-Ti_3_C_2_, and (**c**) PS/O-Ti_3_C_2_ nanocomposites.

The preparation process for exfoliated Ti_3_C_2_ nanosheets, functionalized O-Ti_3_C_2_, and PS/O-Ti_3_C_2_ nanocomposite is shown in Scheme 1.

### 2.5. Characterizations

X-ray diffraction (XRD) patterns were obtained by a Miniflex II (Rigaku) with Cu-Kα radiation (λ = 1.5406 Å) with a 2*θ* range of 3–60° and used to study the crystal structures of Ti_3_AlC_2_, Ti_3_C_2_, and O-Ti_3_C_2_. Atomic force microscopy (AFM) was conducted using a Bruker Dimension Icon AFM. Fourier transform infrared (FTIR) spectrums were collected by a Nicolet iS5 (Thermo Scientific) equipped with iD7 ATR. Scanning electron microscopy (SEM) images were obtained by JEOL JSM-7500F at 5 kV acceleration voltage and used to observe the morphology of Ti_3_AlC_2_, Ti_3_C_2_, O-Ti_3_C_2_, and the fracture surface of the PS nanocomposites. Electron distribution spectroscopy (EDS) results were obtained using the Oxford EDS system at 20 kV acceleration voltage and were used to observe the distribution of Ti and C from O-Ti_3_C_2_ in the PS nanocomposites. Thermogravimetric analysis (TGA) was carried out to investigate the thermal performance of all the samples on a TGA 5500 thermal analyzer (TA Instruments Inc., New Castle, DE, USA) from 30 °C to 700 °C at a heating rate of 20 °C/min under a nitrogen atmosphere. Pyrolysis-combustion flow calorimetric (PCFC) measurements were performed by a microscale combustion calorimeter (MCC, Fire Testing Technology Ltd., Gosport, UK) to investigate the flammability and combustibility of PS/Ti_3_AlC_2_, PS/Ti_3_C_2_, and PS/O-Ti_3_C_2_ nanocomposites. For each run, the pellet-shaped sample was accurately weighed (*ca.* 4.00 mg) and then heated to 900 °C with a heating rate of 1 °C/s. The volatile thermal degradation products were then mixed with a stream of nitrogen (80 cm^3^/min) and oxygen (20 cm^3^/min) before entering a combustion chamber.

## 3. Results and Discussion

### 3.1. Characterizations of O-Ti_3_C_2_ Nanosheets and Its PS Nanocomposites

**Figure 1:**
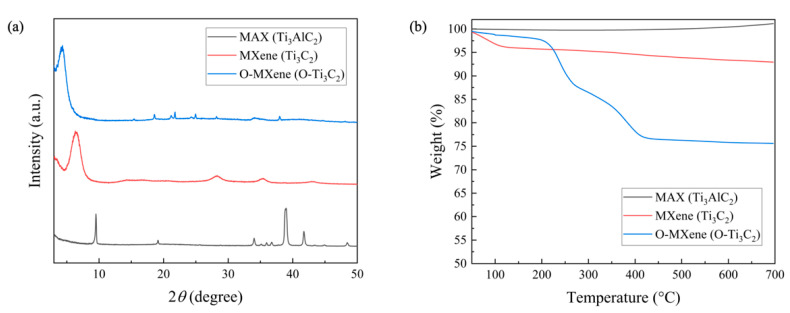
(**a**) XRD patterns of MAX phase (Ti_3_AlC_2_), MXene nanosheets (Ti_3_C_2_), and MXene–organic hybrids (O-Ti_3_C_2_); (**b**) TGA curves of Ti_3_AlC_2_, Ti_3_C_2_, and O-Ti_3_C_2_.

The crystalline phases of bulk Ti_3_AlC_2_, exfoliated Ti_3_C_2_ nanosheets, and the functionalized O-Ti_3_C_2_ nanosheets were studied using X-ray diffraction (XRD). As shown in Figure 1a, (002) and (104) characteristic peaks were observed at around 9.6° and 39.0° in the bulk Ti_3_AlC_2_ (grey line). After a selective etching by LiF and HCl, the disappearance of the diffraction peak at around 39.0° (104) indicates that the aluminum layer was removed from bulk Ti_3_AlC_2_ (red line). In addition, the pronounced peak (002) shifted from a 2*θ* angle of 9.6° to a lower 2*θ* angle of around 6.5°, which is typical for Ti_3_C_2_T_x_ nanosheets [31,32]. The Supplementary Material includes the AFM image (Figure S1) of Ti_3_C_2_ nanosheets, with a thickness of ∼1.6 nm, indicating single-layer nanosheets [33]. Furthermore, the XRD pronounced peak (002) shifts to a lower 2*θ* angle (4.3°) after the organic functionalization (blue line). This phenomenon suggests that the interplanar spacing between the nanosheets increased from 13.4 Å to 20.5 Å due to the alkyl chains in the cationic surfactant (e.g., OTAB), which indicates the successful functionalization of Ti_3_C_2_ nanosheets to O-Ti_3_C_2_ [34]. It has been reported that once the positively charged head (–N(CH_3_)_3_) interacts with the electronegative oxygen atoms in the Ti–O–Ti groups (proved by Figure S2 in the Supplementary Material) on the surface of Ti_3_C_2_ nanosheets, the hydrophobicity of the resulting O-Ti_3_C_2_ is expected to increase [17,35]. This phenomenon could solve the compatibility issue with hydrophobic polymers.

The thermal stabilities of bulk Ti_3_AlC_2_, exfoliated Ti_3_C_2_ nanosheets, and the functionalized O-Ti_3_C_2_ nanosheets were studied by TGA, and the results are shown in Figure 1b. The commercial Ti_3_AlC_2_ is thermally stable under nitrogen (grey line), except for a 1 wt% mass increase in the high-temperature region (higher than 500 °C) due to the selective oxidation of Al into Al_2_O_3_ under a high-purity nitrogen atmosphere [36]. After etching, exfoliated Ti_3_C_2_ nanosheets are still thermally stable until 700 °C (red line), except for a 3 wt% mass loss at around 100 °C and gradual mass loss afterward. The reason for the first mass loss is likely the presence of small amounts of residual water, and the following consecutive mass loss is likely due to the removal of unstable surface functional groups (e.g., -OH, =O, and -F). After functionalization, O-Ti_3_C_2_ exhibits significant mass loss (24.4 wt%) due to the grafting of cationic surfactant OTAB (blue line). The final 75.6 wt% residue indicates that OTAB is functionalized to the surface of Ti_3_C_2_ nanosheets after washing. Details of the calculations are described in the Supplementary Material.

**Figure 2:**
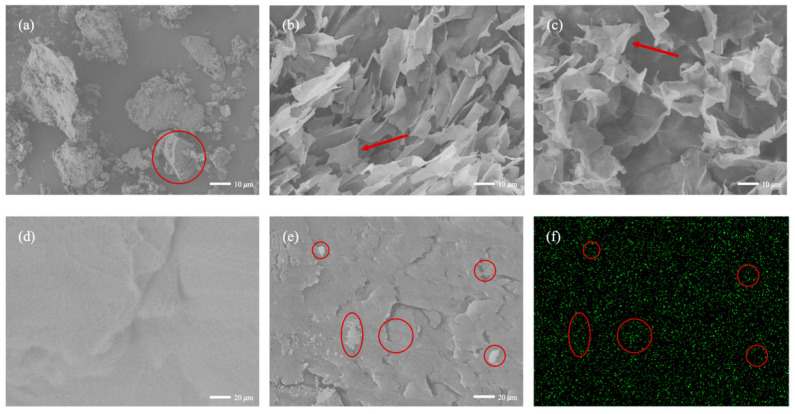
SEM micrograph of (**a**) bulk Ti_3_AlC_2_ powder; (**b**) freeze-dried Ti_3_C_2_ nanosheets; (**c**) freeze-dried O-Ti_3_C_2_ nanosheets; (**d**) PS surface; (**e**) PS/O-Ti_3_C_2_ nanocomposite surface; and (**f**) EDS for Ti element mapping on PS/O-Ti_3_C_2_ nanocomposite surface.

The morphologies of the bulk Ti_3_AlC_2_, exfoliated Ti_3_C_2_ nanosheets, and the functionalized O-Ti_3_C_2_ nanosheets were studied by scanning electron microscopy (SEM), and the results are shown in Figure 2a–c. The SEM micrograph of the Ti_3_AlC_2_ powder (Figure 2a) indicates a different bulk particle structure (circled) than the 2D layer Ti_3_C_2_ nanosheets after successful exfoliation and freeze-drying (Figure 2b, pointed). After the functionalization for O-Ti_3_C_2_ nanosheets, the 2D layered structure is maintained (Figure 2c, pointed).

The dispersion of additives has a significant effect on the flame retardancy performance of nanocomposites, especially for those made of 2D layered nanomaterial fillers, as they tend to agglomerate or restack [8]. Therefore, the dispersion of O-Ti_3_C_2_ nanosheets in the PS matrix was studied by SEM and electron distribution spectroscopy (EDS), as shown in Figure 2d–f. The SEM micrograph of the neat PS surface (Figure 2d) shows a comparatively smooth surface, whereas the SEM micrograph of the nanocomposite surface (Figure 2e) shows a slightly rougher fracture interface of PS surface but no pronounced agglomeration of the O-Ti_3_C_2_ nanosheets, indicating that they are uniformly distributed in the PS matrix. The element mapping (Figure 2f, circled) also suggests a uniform distribution of Ti in the PS matrix. This uniform dispersion indicates that the compatibility issue of hydrophilic MXene with hydrophobic PS was greatly improved by organic functionalization. More detailed calculations (Supplementary Material) proved that O-Ti_3_C_2_ nanosheets were loaded to the PS matrix.

### 3.2. Thermal Stability of PS/O-Ti_3_C_2_

**Figure 3:**
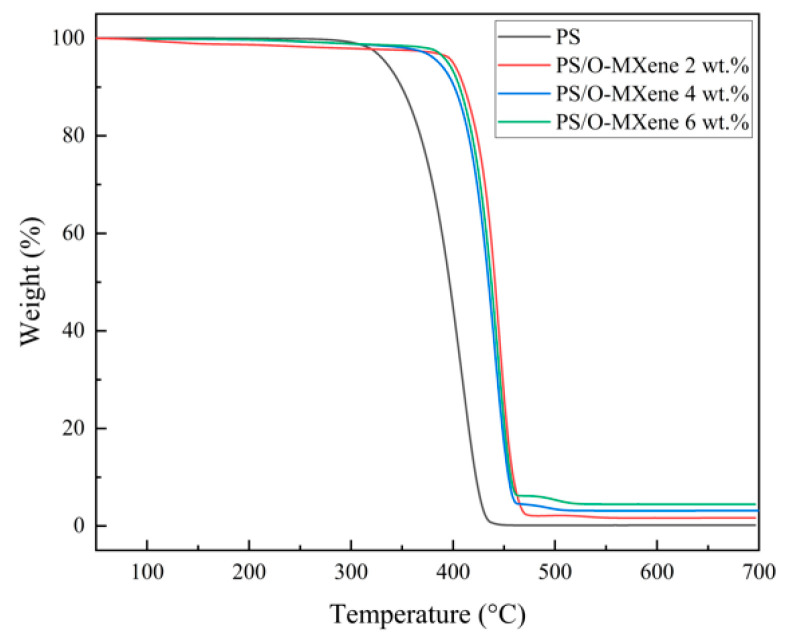
TGA curves of PS and PS/O-Ti_3_C_2_ nanocomposites with different weight ratios.

The thermal decomposition behaviors of PS and PS/O-Ti_3_C_2_ nanocomposites were studied by TGA, and the results are summarized in Table 1 and Figure 3. In this study, the onset decomposition temperature is defined as the temperature at 5 wt% weight loss (T_5%_). PS in our study reached its T_5%_ at 334 °C and the maximum thermal degradation (T_max_) at 410 °C. The thermal degradation of PS is a typical radical chain scission process, which includes initiation, propagation, and termination reactions. Monomer, dimer, and trimer of styrene are the primary thermal decomposition products for neat polystyrene [37], and trace amounts (0.18%) of residues were left at around 430 °C (when the curvature stabilized).

The thermal degradation trends were similar for PS nanocomposite, with the exception that the T_5%_ and T_max_ were delayed. Among all samples, only the nanocomposite with bulk Ti_3_AlC_2_ powder additive is the most similar in thermal performance to neat PS. Furthermore, the nanocomposites with 2D layered Ti_3_C_2_ and O-Ti_3_C_2_ delayed the T_5%_ by 60 °C on average. This was mainly due to the physical barrier effect of the MXene nanosheets with excellent thermal stability, preventing the PS molecular chain from earlier degradation. This phenomenon suggests that the layered MXene nanosheets delay the PS degradation and significantly improve thermal stability.

### 3.3. Flammability Behavior of PS/O-Ti_3_C_2_

**Figure 4:**
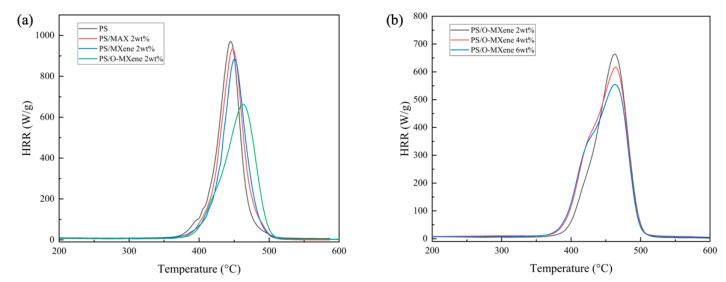
(**a**) PCFC HRR curves of PS and its nanocomposites with same weight ratio; (**b**) PCFC HRR curves of PS/O-Ti_3_C_2_ nanocomposites with different weight ratios.

PCFC was used to investigate the flammability and fire safety properties of the PS nanocomposites, and the results are summarized in Table 1 and Figure 4a. The pHRR of the neat PS is 968.8 W/g, as shown in the HRR vs. temperature curve in Figure 4a. This value is a typical value for PS and within the calibration range provided by the manufacturer of PCFC. When incorporating 2 wt% filler, the PS/Ti_3_AlC_2_ nanocomposite has a similar pHRR (4% in difference) as neat PS, and the PS/Ti_3_C_2_ nanocomposite lowers the pHRR value by 9%. However, the pHRR value reduced considerably (32%) with the PS/O-Ti_3_C_2_ nanocomposite, probably due to the improvement dispersibility of O-Ti_3_C_2_ in DMF. Better dispersion could form a more uniform barrier that could result in a “tortuous path” to isolate the flammable and volatile pyrolysis products from the oxygen supply, slowing down the thermal degradation process and lowering the flammability when combined with the high thermal stability of MXene. Moreover, the combustion of gas products is typically a complete combustion under normal PCFC operation conditions (with oxygen) [38], which means the 32% reduction in pHRR values suggests that there are fewer volatile products transferring to the gas phase.

In addition, we observed (Figure 4a) that the T_pHRR_ of PS/O-Ti_3_C_2_ nanocomposites was shifted to a higher temperature (+22 °C) compared to the neat PS. Another observation is that the THR values for PS/Ti_3_C_2_ and PS/O-Ti_3_C_2_ nanocomposites are just slightly lower than the neat PS, considering the Ti_3_C_2_ and O-Ti_3_C_2_ nanosheet loading. These two phenomena suggest that the pyrolysis process of the PS remains almost unchanged, but the flammable pyrolysis products are released slower. Wilkie et al. also reported a considerable reduction in the pHRR of PS nanocomposites with a layered structure [37]. Since the layered structure in the nanocomposite acts as a barrier to heat and mass transfer, the degradation products are retained for a more extended period, spreading out the degradation over time and lowering the peak value.

**Figure 5:**
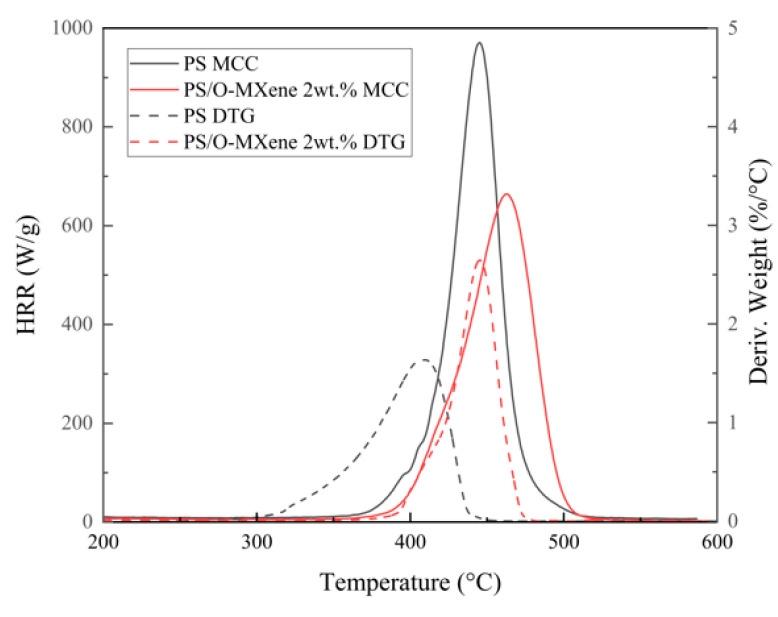
PCFC HRR vs. DTG curves of PS and PS/O-Ti_3_C_2_ nanocomposites.

The reduced pHRR trend continues when increasing the O-Ti_3_C_2_ weight percentage in PS nanocomposites (Figure 4b). For example, when O-Ti_3_C_2_ was 6 wt% in the PS matrix, the pHRR of the nanocomposite was reduced by 43%. However, the T_pHRR_ was not shifted further, which suggests the T_pHRR_ shifted effect may not be related to filler concentration but rather to the unique chemical structure of O-Ti_3_C_2_. In addition, the first derivatives of the TGA curve (the DTG curve) and HRR curve from PCFC for PS and PS/O-Ti_3_C_2_ are shown in the same plot as Figure 5. The solid line represents the heat-related HRR curve, and the dashed line represents the mass-related DTG curve. The temperature at the maximum mass loss in the DTG curve for PS/O-Ti_3_C_2_ nanocomposites (with 2 wt% filler) shifting higher compared to the neat PS sample shows a similar trend with HRR curves, which means the nanocomposites postpone the polymer degradation.

As long as the heating rate (1 °C/s in this study) remains constant throughout the test, HRC could be used as a good predictor of flammability, regardless of the shape or mass of the samples [26,39]. It has been found that the MXene–organic hybrid is an excellent candidate for reducing HRC, implying lower flammability with a lower risk of fire hazards. In addition, the reduction in HRC is defined by 100 × (HRC_neat polymer_ − HRC_nanocomposite_)/HRC_neat polymer_, and the reduction in HRC for PS/O-Ti_3_C_2_ with 2/4/6 wt% filler is 32/37/44%. The comparison of the reduction in HRC indicates that increasing the amount of O-Ti_3_C_2_ in the PS matrix continues lowering the flammability. As a result, the enhanced thermal stability and flammability performance are ascribed to the amount of O-Ti_3_C_2_ in the nanocomposite, which slows down the degradation process on the PS nanocomposite system via the barrier effect.

**Figure 6:**
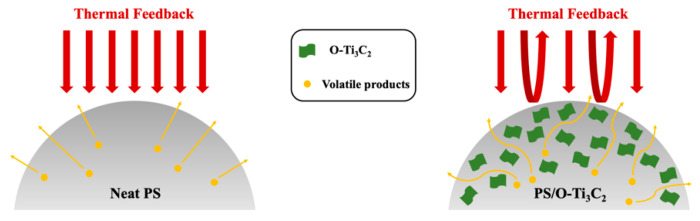
Proposed mechanisms of reduced flammability of PS/O-Ti_3_C_2_ nanocomposites.

According to the results above, the enhanced thermal stability and flammability performance are the results of the O-Ti_3_C_2_ presented in the PS nanocomposite. Figure 6 illustrates the proposed mechanisms for the slower degradation of PS nanocomposites. In the neat PS, a significant amount of volatile gas was produced due to the rapid decomposition of polystyrene. In addition, due to the poor distribution of MXene in hydrophobic polymers, the decomposition process is similar in PS/Ti_3_C_2_ nanocomposite. However, when O-Ti_3_C_2_ with high thermal stability is introduced to PS nanocomposite, the layered structure of MXene–organic hybrid nanosheets could benefit from improving the 2D thermal- and mass-transfer barrier effect. It would impede heat transfer and retard the transfer of pyrolysis gas products. The barrier effect becomes more prominent as the O-Ti_3_C_2_ nanosheets increase.

## 4. Conclusions

In this study, Ti_3_C_2_ nanosheets were successfully exfoliated from Ti_3_AlC_2_ powder and functionalized by cationic surfactant OTAB to enhance dispersion in PS to produce a nanocomposite with improved thermal stability and flammability performance compared to neat PS. In addition, the nanosheet functionalizations increased the interlayer distance between the nanosheets and enhanced its dispersion in the PS matrix. According to the PCFC results, the PS/O-Ti_3_C_2_ nanocomposites (with 2 wt% filler) effectively lower the flammability (lower the pHRR by 32%) and significantly shift the T_pHRR_ to a higher temperature by 22 °C. The comparison between the PS/bulk MAX and different weight ratios of PS/O-Ti_3_C_2_ nanocomposites reveals that the thermal stability and 2D thermal- and mass-transfer barrier effect of MXene–organic hybrid nanosheets play essential roles in delaying the polymer degradation. Hence, this work demonstrates a straightforward method to prepare MXene–organic hybrids, which have the potential to be promising FR additives in PS nanocomposites. In the future, the synergistic effect with other FRs to improve flame retardancy as well as the application of different MXene types as filler should be examined.

## Figures and Tables

**Table 1:** TGA and PCFC results of PS and its different nanocomposites.

Sample	TGA Result			PCFC Results			
	T_5%_ (°C)	T_max_ (°C)	Residues (wt%)	THR (kJ/g)	pHRR (W/g)	HRC (J/g∙K)	T_pHRR_ (°C)
Neat PS	334	410	0.18	39.9	968	991	441
PS/Ti_3_AlC_2_ 2 wt%	339	418	2.25	39.8	931	939	448
PS/Ti_3_C_2_ 2 wt%	397	429	2.24	37.5	878	894	451
PS/O-Ti_3_C_2_ 2 wt%	399	445	1.66	37.3	660	673	463
PS/O-Ti_3_C_2_ 4 wt%	386	441	3.15	37.9	608	622	464
PS/O-Ti_3_C_2_ 6 wt%	395	444	4.46	37.6	548	560	463

## Data Availability

Not applicable.

## Supplementary Materials

The following are available online at https://www.mdpi.com/article/10.3390/polym14061213/s1, Figure S1: AFM image of Ti_3_C_2_ dropcast dispersion. Figure S2: FTIR patterns of MAX Ti_3_AlC_2_ and MXene Ti_3_C_2_T_x_. Figure S3: Dispersion of MXene nanosheets and MXene-organic hybrids in water and DMF. Figure S4: TGA and DTG curves of OTAB under nitrogen conditions. Figure S5: EDS element distribution and signal intensity. Table S1: Electron distribution spectroscopy EDS result for weight%.
