# Supplementary material for: Thermal Stability and Flammability Studies of MXene–Organic Hybrid Polystyrene Nanocomposites
Source: Polymers (Basel). 2022 Mar 17;14(6):1213. doi: 10.3390/polym14061213 (PMC8954563; doi:10.3390/polym14061213)
Supplement: Supplementary file 1 [file polymers-14-01213-s001.zip › polymers-1633539-supplementary.pdf]

polymers

MDPI

Supplementary Material

Thermal Stability and Flammability Studies of MXene-Organic Hybrid Polystyrene Nanocomposites

Zhuoran Zhang [1], Huaixuan Cao [1,2], Yufeng Quan [1], Rong Ma [1], Emily B. Pentzer [2,3], Micah J. Green [1,2], and Qingsheng Wang [1,*]

1 Artie McFerrin Department of Chemical Engineering, Texas A&M University, College Station, TX 77843, USA; zhuoranzhang@tamu.edu (Z.Z.); chx744933979@tamu.edu (H.C.); yquan@tamu.edu (Y.Q.); marong1863@tamu.edu (R.M.); micah.green@tamu.edu (M.J.G.)
2 Department of Materials Science and Engineering, Texas A&M University, College Station, TX 77843, USA; emilypentzer@chem.tamu.edu (E.B.P.)
3 Department of Chemistry, Texas A&M University, College Station, TX 77843, USA
* Correspondence: qwang@tamu.edu

Citation: Zhang, Z.; Cao, H.; Quan, Y.; Ma, R.; Pentzer, E.B.; Green, M.J.; Wang, Q. Thermal Stability and Flammability Studies of MXene–Organic Hybrid Polystyrene Nanocomposites. *Polymers* **2022**, *14*, 1213. https://doi.org/10.3390/polym14061213

Academic Editor: Editors: Francisco Javier Espinach Orús and Quim Tarrés Farrés

Received: 25 February 2022
Accepted: 14 March 2022
Published: 17 March 2022

Publisher's Note: MDPI stays neutral with regard to jurisdictional claims in published maps and institutional affiliations.

Copyright: © 2022 by the authors. Submitted for possible open access publication under the terms and conditions of the Creative Commons Attribution (CC BY) license (https://creativecommons.org/licenses/by/4.0/).

1. Characterizations of MAX (Ti_3AlC_2), MXene (Ti_3C_2), and O-Ti_3C_2

Figure S1 shows the atomic force microscopy (AFM) image of Ti_3C_2 nanosheets, with a thickness of ~1.6 nm, indicating single-layer nanosheets.

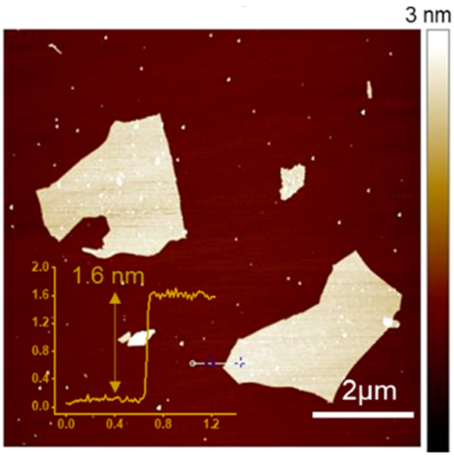

Figure S1. AFM image of Ti_3C_2 dropcast dispersion (inset shows the thickness of the Ti_3C_2 nanosheet along the line).

Fourier transform infrared spectroscopy (FTIR) patterns of $Ti_3C_2T_x$ MXene are shown in **Figure S2**. It can be observed that the additional Ti–OH (3369 cm^{-1}), C–F (1142 cm^{-1}), and Ti–O (623 cm^{-1}) groups are introduced into $Ti_3C_2T_x$.

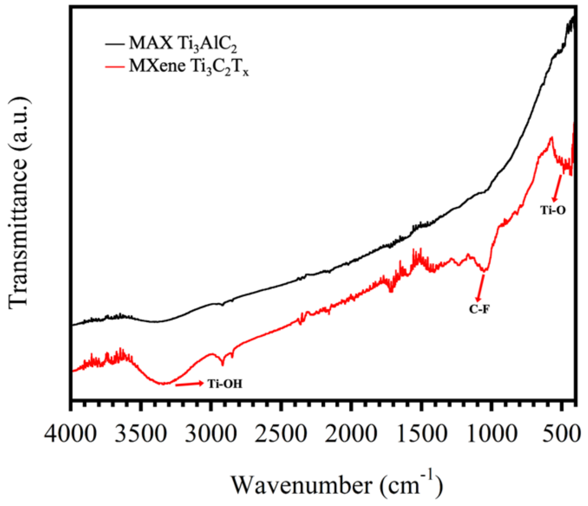

Figure S2. FTIR patterns of MAX Ti_3AlC_2 and MXene $Ti_3C_2T_x$

2. Dispersion of O-Ti_3C_2 in DMF

It has been reported that once the positively charged head (–N(CH_3)$_3$) interacts with the electronegative oxygen atoms in the Ti–O–Ti groups on Ti_3C_2 monolayer, the hydrophobicity of the resulting MXene-organic hybrids is expected to increase. We can observe (**Figure S3**) after 24 h: 1) the MXene nanosheets can form a good dispersion in DI water; 2) the dispersion is poor in DMF which could cause a poor dispersion in PS nanocomposites and therefore influence the flame retardancy; 3) the modified MXene-organic hybrids is not dispersed in DI water due to its charged head; 4) and the dispersion in DMF is homogeneous and stable, which could lead to a better dispersion in PS nanocomposite synthesis.

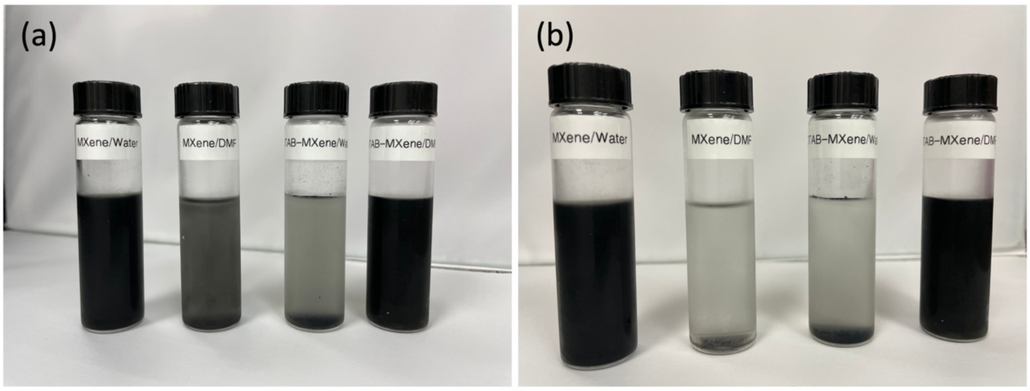

Figure S3. Dispersion of MXene nanosheets and MXene-organic hybrids in water and DMF (from left to right: MXene/DI water, MXene/DMF, O-MXene/DI water, O-MXene/DMF) for (**a**) 0 h, (**b**) 24 h.

3. O-Ti_3C_2 TGA Result Calculation

The thermal stability of OTAB was studied by TGA and DTG curves, and the results are shown in **Figure S4**. The initial degradation temperatures for OTAB at 5 wt% was 230 °C, and the OTAB cationic surfactant was completely degraded at around 280 °C. For the modified O-Ti_3C_2, the initial degradation onset temperature at 5 wt% was also 230 °C, which indicates the major

thermal degradation is due to the grafting of cationic surfactant OTAB. As mentioned in the experimental section, 5.25 mL of organic cationic modifier OTAB/deionized water (DI) solution (1 wt%) was added to 150 mL MXene dispersion (1 mg/mL). Theoretically, 74.1 wt% of the O-Ti_3C_2 should be thermally stable Ti_3C_2. Considering the mass loss due to water and surface functional groups, the final 75.6 wt% residue indicates OTAB is successfully attached to the surface of Ti_3C_2 nanosheets.

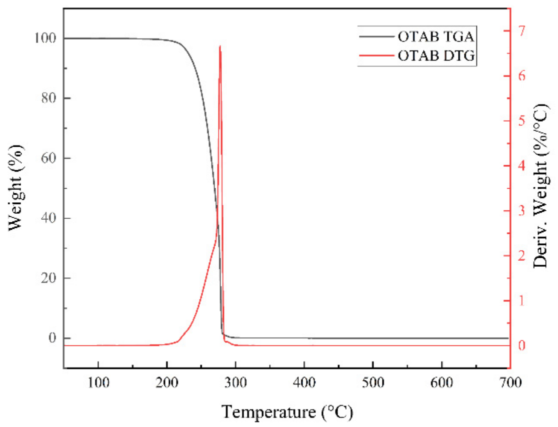

Figure S4. TGA and DTG curves of OTAB under nitrogen conditions

4. PS/O-Ti_3C_2 (2 wt%) EDS Result Calculation

The detailed electron distribution spectroscopy (EDS) results were shown in **Table S1** and **Figure S5**. Except for the C from the carbon bones of PS and the carbon layer of MXene, the Ti element is a characteristic signal in EDS. The theoretical weight% of the Ti element in the nanocomposite is around 1.2%. The observed Ti element in our nanocomposites (0.89%) indicates the successful synthesis of PS/O-Ti_3C_2 nanocomposite.

Table S1. Electron distribution spectroscopy EDS result for weight%.

Element	Weight%	Atomic%
C	99.11	99.78
Ti	0.89	0.22
Total	100.00	

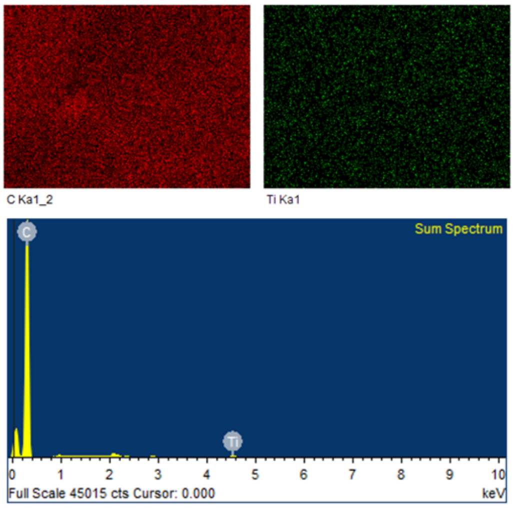

Figure S5. EDS element distribution and signal intensity
